# Using Applied Conversation Analysis in Medical Education

**DOI:** 10.1093/geroni/igaa057.2403

**Published:** 2020-12-16

**Authors:** Sean Halpin, Kathryn Roulston, Michael Konomos

**Affiliations:** 1 University of Georgia, Decatur, Georgia, United States; 2 University of Georgia, Athens, Georgia, United States; 3 Emory University, Atlanta, Georgia, United States

## Abstract

Successful implementation of patient medical education is contingent on the communication strategies used by nurses, patients, and caregivers. Applied conversation analysis (A-CA) is a sociological and linguistic technique aimed at understanding how interaction is accomplished. In this demonstration of A-CA, the authors draw on an 18-month iterative-formative evaluation of patient education that precedes autologous stem cell transplant for persons diagnosed with multiple myeloma (N=70), a type of cancer which disproportionately impacts older adults. In this study, patients and caregivers received supplemental education videos before their formal education session with a nurse coordinator. Using A-CA, we examined how nurses, patients, and caregivers orient toward the videos; including demonstrated knowledge by patients and caregivers. Nurses justified repeating topics from the videos. Through a focus on the function that language plays in sequences of interaction, it may be possible to determine strategies for improving patient education, and, consequently positively impact patient care..

